# Cultural Differences in Fear of Negative Evaluation After Social Norm Transgressions and the Impact on Mental Health

**DOI:** 10.3389/fpsyg.2022.804841

**Published:** 2022-04-13

**Authors:** Mamta Vaswani, Victoria M. Esses, Ian R. Newby-Clark, Benjamin Giguère

**Affiliations:** ^1^Network for Economic and Social Trends (NEST), University of Western Ontario, London, ON, Canada; ^2^Department of Psychology, University of Guelph, Guelph, ON, Canada

**Keywords:** cross-cultural differences, social norms, fear, evaluation, mental health, distress

## Abstract

Social norm transgressions are assumed to be at the root of numerous substantial negative outcomes for transgressors. There is a prevailing notion among lay people and scholars that transgressing social norms can negatively impact one’s mental health. The present research aimed to examine this assumption, focusing on clinically relevant outcomes such as anxiety and depression. The present research further aimed to examine a social cognitive process for these outcomes in the form of fear of negative evaluations as a result of one’s norm transgressing behavior. Specifically, it examined whether it is negative evaluations about ourselves or about those close to us that mediates the effect of social norm transgressions, and whether those may vary as a function of culture. Results of the present research, including a study with a community sample (*N* = 410), suggest a positive association between social norm transgressions and psychological distress. Results also suggest that increased fear of negative evaluation mediates that association but does so differently for people from more collectivistic cultures and people from less collectivistic cultures. For people from more collectivistic cultures increased fear of negative evaluation of close others may mediate the association between social norm transgressions and psychological distress. However, for people from less collectivistic cultures that association may be mediated by increased fear of negative evaluation of oneself. Implications for research on consequences of social norm transgressions and cross-cultural differences in perceptions of such consequences are discussed as are practical implications for motivating social norm adherence and the maintenance of constructive social norms.

## Introduction

Social norms are commonly agreed upon informal rules or guidelines for human behavior (Turner, 1991). Adhering to social norms maintains agreed upon standards about what are right and proper ways of thinking, feeling, and behaving (Turner, 1991). When people adhere to social norms, they typically receive positive social responses such as approval from others (Turner, 1991; Cialdini and Trost, 1998). However, when people behave in a manner that transgresses social norms, and which can threaten desirable standards, they may suffer negative social responses such as disapproval from others (Turner, 1991; Cialdini and Trost, 1998). As such, lay people and scholars assume that transgressing social norms contributes negatively to psychological well-being, in part, because transgressors are concerned about negative evaluations from others. The aim of the present research was to examine these common assumptions about the consequences of social norm transgressions.

Given that people may be concerned about their social image and the social image of people close to them, we also distinguished between fear that social norm transgressions would lead to negative evaluations of oneself and fear that social norm transgressions would lead to negative evaluations of close others, and whether the extent of this distinction varies as a function of one’s cultural background. Specifically, people from more collectivistic cultures (e.g., Eastern cultures) may be especially impacted by concerns about their norm transgressions reflecting on close others, whereas people from less collectivistic cultures (e.g., Western cultures) may be especially impacted by concerns about their norm transgressions reflecting on themselves. Such a distinction is rooted in the notion that people from more collectivistic cultures tend to be highly attuned to the social image of close others as compared to people from less collectivistic cultures (Uskul et al., 2012; Rodriguez Mosquera et al., 2014). Therefore, cross-cultural differences in the role of fear of negative evaluation in mediating between social norm transgression and mental health was also examined.

### Social Norm Transgressions, Fear of Negative Evaluation, and Psychological Distress

Social norms are generally agreed upon and adopted informal rules or guidelines that communicate what is considered appropriate ways of thinking, feeling, and behaving versus what is considered inappropriate (Turner, 1991). When people engage in behavior that aligns with social norms, they maintain agreed upon standards about what is right and proper, and can expect to receive positive social responses such as approval from others (Turner, 1991; Cialdini and Trost, 1998). However, when people engage in behavior that transgresses social norms, they threaten those standards, and can expect to receive negative social responses such as disapproval from others (Festinger, 1950; Turner, 1991; Cialdini and Trost, 1998). Norm violations have been found to result in less favorable evaluations and dislike of norm transgressors (e.g., Levine et al., 1976; Arnold and Greenberg, 1980; Abrams et al., 2002). In addition, social norm transgressions can result in social rejection of norm transgressors (e.g., Schachter, 1951; Schachter et al., 1954; Berkowitz and Howard, 1959). Therefore, transgressing social norms can elicit a variety of negative social responses from others.

At an intrapersonal level, people who transgress social norms might respond to their own transgressions in various ways (Aronfreed, 1961). For instance, following social norm transgressions people may anticipate and perceive experiencing social rejection, experience negative emotions such as guilt and shame, and may engage in subsequent problematic behavior that further transgresses social norms (e.g., McGraw, 1987; Giguère et al., 2014; Vaswani et al., 2021). Another intrapersonal response to having transgressed social norms could be *fear of negative evaluation* (Watson and Friend, 1969). Fear of negative evaluation includes expectations of, and concern and distress over, being evaluated negatively (Watson and Friend, 1969; Smith and Sarason, 1975; Leary, 1983). Given that social norm transgressions often result in negative social consequences from other people (e.g., negative evaluations, dislike, disapproval, social rejection), norm transgressors may be concerned about others’ perceptions of them. As such, after having transgressed social norms people could have elevated fears of being negatively evaluated (e.g., Giguère et al., 2016).

Fear of negative evaluation after having transgressed social norms may, in turn, be associated with psychological distress such as anxiety, depression, and stress. Indeed, the association between various forms of evaluative anxiety, and more general anxiety, depression, and stress has been well-established (e.g., Schneier et al., 1992; Kessler et al., 2005; Haines et al., 2016; see Clark et al., 1997). Furthermore, Pagliaro et al. (2016) found support for an association between (immoral) norm transgressing behavior and threatening feelings including anxiety and stress, an association that was mediated by anticipated negative responses (less respect) from others. Therefore, the present research aimed to examine the mediating role of fear of negative evaluation in the association between transgressing social norms and psychological distress.

### Cultural Differences in Fear of Negative Evaluation After Social Norm Transgressions

Although people from different cultural groups share many values, beliefs, and social norms, there also exist some differences that influence how people think, feel, and behave. One such differences is the extent to which people are more strongly integrated into cohesive groups, *collectivism* (Hofstede, 2011). People from more collectivistic cultures, such as Eastern cultures, tend to be more connected to others and group dependent than are people from less collectivistic cultures, such as Western cultures (Markus and Kitayama, 1991; Triandis, 1995; Hofstede, 2011). For instance, children from collectivistic cultures are socialized to be interdependent with their families, to feel a sense of obligation to their families, and to value the greater good of the family unit over their personal gains (see Kwak, 2003; Stuart and Ward, 2011; Heine, 2012). Being highly connected to their families, people from collectivistic cultures tend to think and behave in a manner that aligns closely with the expectations of their families (e.g., Lalonde et al., 2004, 2013; Uskul et al., 2007; Lou et al., 2012).

Given their interdependent nature, people from more collectivistic cultures tend to be more attuned to the social image of close others than are people from less collectivistic cultures (Rodriguez Mosquera et al., 2002, 2008, 2014; Uskul et al., 2012). Such a focus is learned at an early age. Children from collectivistic cultures are socialized to be concerned about how their actions may impact their families, and their poor behavior is seen as tarnishing their families’ social image (e.g., Munniksma et al., 2012; Uskul et al., 2012). As such, one’s social image can be closely connected to the social image of close others. Indeed, Rodriguez Mosquera et al. (2014) found that when close others’ social images are threatened, people from more collectivistic cultures are more likely to feel their own social image may also be threatened than people from less collectivistic cultures. Paralleling the impact of collectivistic cultures’ interdependent nature on perceptions of interconnected social images, Fortune and Newby-Clark (2008) found that being socially and physically close to others tends to result in (erroneous) perceptions that negative evaluations of those others will also be extended to oneself.

In addition to one’s own and close others’ social images being interconnected, consequences of tarnished social images are also interconnected for people from collectivistic cultures. For instance, people from more collectivistic cultures are more likely to anticipate negative emotional consequences for themselves as well as their close others as a result of their social image being tarnished, whereas people from less collectivistic cultures are more likely to anticipate negative emotional consequences more so for themselves (Uskul et al., 2012). Similarly, when close others’ social images are threatened, people from more collectivistic cultures are more likely to feel negative emotions such as shame than people from less collectivistic cultures (Rodriguez Mosquera et al., 2002). Rodriguez Mosquera et al. (2014) have also found that people from more collectivistic cultures experience negative emotions equally when their social image and when close others’ social images are threatened; however, people from less collectivistic cultures tend to experience negative emotions more so when their own social image is threatened.

In sum, people from more collectivistic cultures tend to be highly attuned to the social image of close others, concerned about their tarnished social images negatively impacting close others, and negatively impacted by the tarnished social image of close others. This contrasts with people from less collectivistic cultures who tend to be more attuned to their own social image, concerned about their tarnished social images negatively impacting themselves, and negatively impacted by their own tarnished social images. Based on such cultural differences, we suggest that people from more collectivistic cultures may experience psychological distress after having transgressed social norms by way of fear over the negative evaluations of close others. This is in contrast to people from less collectivistic cultures who may be more likely to experience psychological distress after having transgressed social norms by way of fear over negative evaluations of themselves. As such, the present research aimed to examine cultural differences in fear of negative evaluation of the self and close others as an avenue through which social norm transgressions are associated with psychological distress.

### Present Research

The aims of the present research were to examine the association between social norm transgressions and psychological distress, and cultural differences in the type of fear of negative evaluation that may mediate that association: fear of negative evaluation of close others for those from more collectivistic cultures and fear of negative evaluation of the self for those from less collectivistic cultures.

## Study 1

Study 1 was a pilot study conducted as part of a larger study on social norm transgressions in order determine what events are commonly reported as social norm transgressions. The resulting commonly reported events were then used in Study 2 in order to examine our hypotheses.

### Method

#### Participants

Seventy undergraduate students were recruited from a university in Southwestern Ontario, Canada to take part in exchange for course credit. Participants (14 male, 56 female) ranged in age between 18 and 42 years (*M* = 19.17, *SD* = 3.03). Most participants self-identified as White (*N* = 48), six as East Asian, four as South Asian, four as Middle Eastern, four as Latin American, one as Black, and one as Indigenous.

#### Procedure

After providing consent, participants were asked to complete a questionnaire which began with demographics questions. Participants were then asked to recall and describe a time when they had transgressed a social norm, positioned as something that they would not have wanted their parents to know about. Participants were then debriefed, dismissed, and compensated with their course credit through the university’s electronic participant pool system.

### Results and Discussion

A content analysis of the recalled norm transgression events was conducted. Most norm transgressions recalled involved consuming alcohol (30%) and partying (29%). Others involved promiscuity and dating (17%), using or being in the presence of drugs (9%), lying about grades (9%), stealing (7%), smoking cigarettes or cigars (6%), traffic violations (6%), missing school (4%), fake identification (3%), and harming or harassing others (1%).

The aim of Study 1 was to determine what events were common social norm transgressions so that those events could be used in Study 2 to examine the specific hypotheses of the present research. Of the recalled social norm transgressions, most involved consuming alcohol and partying.

## Study 2

Study 2 aimed to examine the association between social norm transgressions and psychological distress, and cultural differences in the type of fear of negative evaluation that may mediate that association: fear of negative evaluation of close others and fear of negative evaluation of the self. Hypothesis 1 was that social norms transgressions are associated with psychological distress. Hypothesis 2 was that for those from more collectivistic cultures, fear of negative evaluation of close others would mediate the association between social norm transgressions and psychological distress, whereas for those from less collectivistic cultures, fear of negative evaluation of the self would mediate the association between social norm transgressions and psychological distress.

### Method

#### Participants

A community sample of 410 participants was recruited using the services of a market research firm from the Greater Toronto Area in Ontario, Canada. Participants (170 males, 239 females, one other) ranged in age between 18 and 40 years old (*M* = 28.55, *SD* = 6.09). Approximately half of the participants self-identified as South Asian (*N* = 202), representing a cultural group that tends to be more collectivistic, and the other half as White (*N* = 208), representing a cultural group that tends to be less collectivistic.

#### Procedure

After providing consent, participants were asked to complete a questionnaire which began with demographics questions and a measure of collectivism. Given that partying and consuming alcohol, which commonly occurs in party environments, were what most participants had recalled and described as social norm transgressions in Study 1, participants were asked to recall and describe a time when they had partied. Next, to determine how much they felt they had transgressed a social norm, participants were asked to complete a measure of how conflicted they were over what they had done and what they should have done in the event they had described. Participants were then asked to complete measures of fear of negative evaluation of themselves and of their parents as a result of their behavior in the event. Participants were also asked to complete measures of psychological distress before being debriefed and dismissed. Participants were compensated based on the previously established arrangement they had with the market research firm.

#### Measures

##### Collectivism

Participants were asked to complete the Collectivism Scale by Triandis and Gelfand (1998) rated on a 5-point Likert scale ranging from 1 (*strongly disagree*) to 5 (*strongly agree*) (e.g., “It is important to me that I respect the decisions made by my groups.”). Ratings were averaged across items to create combined scores (Cronbach’s *α* South Asian = 0.86, Cronbach’s *α* White = 0.77). Higher values are indicative of higher collectivism.

##### Social Norm Transgression

Participants were asked to complete items adapted from the Norm-Impulse Conflict Scale by Giguère and Lalonde (2010) rated on a 5-point Likert scale ranging from 1 (*strongly disagree*) to 5 (*strongly agree*) (e.g., “I had a clear sense that what I was doing was inappropriate.”). Ratings were averaged across items to create combined scores (Cronbach’s *α* South Asian = 0.85, Cronbach’s *α* White = 0.88). Higher values are indicative of higher perceptions of having transgressed a social norm.

##### Fear of Negative Evaluation of Oneself

Participants were asked to complete items adapted from the Brief Fear of Negative Evaluation Scale by Leary (1983) rated on a 5-point Likert scale ranging from 1 (*strongly disagree*) to 5 (*strongly agree*) (e.g., “Other people’s opinions of me did not bother me.”). Ratings were averaged across items to create combined scores (Cronbach’s *α* South Asian = 0.83, Cronbach’s *α* White = 0.89). Higher values are indicative of higher fear of own negative evaluation.

##### Fear of Negative Evaluation of One’s Parents

Again, participants were asked to complete items adapted from the Brief Fear of Negative Evaluation Scale by Leary (1983) rated on a 5-point Likert scale ranging from 1 (*strongly disagree*) to 5 (*strongly agree*) (e.g., “Other people’s opinions of my parents did not bother me.”). Ratings were averaged across items to create combined scores (Cronbach’s *α* South Asian = 0.73, Cronbach’s *α* White = 0.77). Higher values are indicative of higher fear of parental negative evaluation.

##### Depression

Participants were asked to complete the Depression subscale by Lovibond and Lovibond (1995) rated on a 4-point Likert scale ranging from 0 (*did not apply to me at all*) to 3 (*applied to me very much or most of the time*) (e.g., “I felt that I had nothing to look forward to.”). Ratings were summed and multiplied by two, as is typically done when the measure is used in a clinical setting, to create combined scores ranging from 0 to 42 (Cronbach’s *α* South Asian = 0.94, Cronbach’s *α* White = 0.94). Higher values are indicative of greater depression. In clinical settings depression scores ranging from 0 to 9 are considered normal, 10 to 13 are mild, 14 to 20 are moderate, 21 to 27 are severe, and 28 and above are extremely severe (Lovibond and Lovibond, 1995).

##### Anxiety

Participants were asked to complete the Anxiety subscale by Lovibond and Lovibond (1995) rated on a 4-point Likert scale ranging from 0 (*did not apply to me at all*) to 3 (*applied to me very much or most of the time*) (e.g., “I felt I was close to panic.”). Ratings were summed and multiplied by two, as is typically done when the measure is used in a clinical setting, to create combined scores ranging from 0 to 42 (Cronbach’s *α* South Asian = 0.94, Cronbach’s *α* White = 0.92). Higher values are indicative of greater anxiety. In clinical settings anxiety scores ranging from 0 to 7 are considered normal, 8 to 9 are mild, 10 to 14 are moderate, 15 to 19 are severe, and 20 and above are extremely severe (Lovibond and Lovibond, 1995).

##### Stress

Participants were asked to complete the Stress subscale by Lovibond and Lovibond (1995) rated on a 4-point Likert scale ranging from 0 (*did not apply to me at all*) to 3 (*applied to me very much or most of the time*) (e.g., “I found it hard to wind down.”). Ratings were summed and multiplied by two, as is typically done when the measure is used in a clinical setting, to create combined scores ranging from 0 to 42 (Cronbach’s *α* South Asian = 0.94, Cronbach’s *α* White = 0.92). Higher values are indicative of greater stress. In clinical settings, stress scores ranging from 0 to 14 are considered normal, 15 to 18 are mild, 19 to 25 are moderate, 26 to 33 are severe, and 34 and above are extremely severe (Lovibond and Lovibond, 1995).

### Results

To assess whether the South Asian group represented a more collectivistic cultural group than the White group, a *t*-test was conducted to examine differences between the two groups on the measure of collectivism. On average the South Asian group scored higher on the measure of collectivistic (*M* = 3.89, *SD* = 0.65) than the White group (*M* = 3.77, *SD* = 0.54), *t*(408) = −2.07, *SE* = 0.06, *p* < 0.05, 95% CI for mean difference [−0.24, −0.01].^1^^,^^2^

For statistical completeness, Tables 1 and 2 present descriptive statistics and Pearson correlations for the variables used to test the specific hypotheses: perceptions of having transgressed a social norm, fear of own negative evaluation, fear of parental negative evaluation, depression, anxiety, and stress for each of the South Asian group and the White group. In order to test Hypotheses 1 and 2, two sets of mediation analyses were conducted using the SPSS PROCESS macro (Model 4; Hayes, 2013) entering social norm transgression as the predictor variable and both fear of own negative evaluation and fear of parental negative evaluation as simultaneous mediators for each of depression, anxiety, and stress: one set for the South Asian participants and the other set for the White participants.^3^^,^^4^

**Table 1:** Descriptive statistics and Pearson correlations for perceptions of having transgressed a social norm (SNT), fear of own negative evaluation (FONE), fear of parental negative evaluation (FPNE), depression (DEP), anxiety (ANX), and stress (STR) for the South Asian group, with bootstrapped 95% confidence intervals based on 10,000 bootstrap samples.

Measure	1	2	3	4	5	6
1. SNT	–	0.40^***^ [0.26, 0.52]	0.55^***^ [0.43, 0.66]	0.53^***^ [0.41, 0.63]	0.54^***^ [0.44, 0.64]	0.55^***^ [0.44, 0.65]
2. FONE	–	–	0.51^***^ [0.36, 0.64]	0.36^***^ [0.25, 0.48]	0.30^***^ [0.20, 0.41]	0.36^***^ [0.25, 0.46]
3. FPNE	–	–	–	0.48^***^ [0.37, 0.57]	0.43^***^ [0.33, 0.53]	0.44^***^ [0.33, 0.54]
4. DEP	–	–	–	–	0.89^***^ [0.85, 0.93]	0.92^***^ [0.89, 0.94]
5. ANX	–	–	–	–	–	0.93^***^ [0.90, 0.95]
6. STR	–	–	–	–	–	–
*M*	2.54 [2.41, 2.67]	2.95 [2.84, 3.06]	2.73 [2.63, 2.83]	11.29 [9.71, 12.94]	10.67 [9.07, 12.29]	11.31 [9.75, 12.89]
*SD*	0.94	0.78	0.68	11.91	11.59	11.38

^*^p < 0.05, ^**^p < 0.01, ^***^p < 0.001.

**Table 2:** Descriptive statistics and Pearson correlations for perceptions of having transgressed a social norm (SNT), fear of own negative evaluation (FONE), fear of parental negative evaluation (FPNE), depression (DEP), anxiety (ANX), and stress (STR) for the White group, with bootstrapped 95% confidence intervals based on 10,000 bootstrap samples.

Measure	1	2	3	4	5	6
1. SNT	–	0.26^***^ [0.13, 0.39]	0.57^***^ [0.46, 0.67]	0.36^***^ [0.23, 0.50]	0.46^***^ [0.34, 0.57]	0.38^***^ [0.25, 0.51]
2. FONE	–	–	0.19^**^ [0.04, 0.33]	0.31^***^ [0.18, 0.44]	0.24^***^ [0.12, 0.36]	0.37^***^ [0.27, 0.48]
3. FPNE	–	–	–	0.32^***^ [0.17, 0.46]	0.40^***^ [0.27, 0.52]	0.32^***^ [0.18, 0.44]
4. DEP	–	–	–	–	0.84^***^ [0.77, 0.90]	0.89^***^ [0.85, 0.92]
5. ANX	–	–	–	–	–	0.88^***^ [0.82, 0.92]
6. STR	–	–	–	–	–	–
*M*	2.26 [2.13, 2.38]	3.04 [2.92, 3.15]	2.41 [2.31, 2.50]	10.14 [8.59, 11.72]	8.90 [7.45, 10.34]	11.09 [9.59, 12.56]
*SD*	0.93	0.85	0.70	11.59	10.59	11.05

^*^p < 0.05, ^**^p < 0.01, ^***^p < 0.001.

#### Depression

The estimate reflecting the direct effect of a social norm transgression on depression for the South Asian participants fell within a positive confidence interval [*b* = 4.58, *SE* = 0.89, *p* < 0.001, 95% CI (2.83, 6.33)] as did that estimate for the White participants [*b* = 2.76, *SE* = 0.97, *p* < 0.01, 95% CI (0.84, 4.67)]. These results suggest that as perceptions of transgressing a social norm increase so may depression, providing support for Hypothesis 1.

**Figure 1:**
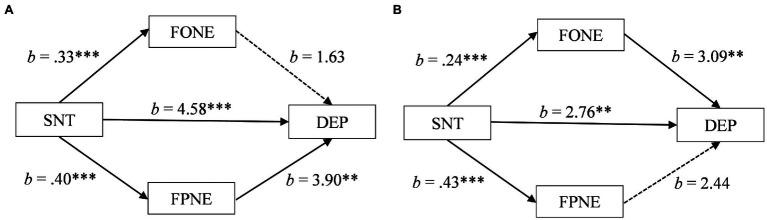
**(A)** Mediation of social norm transgression (SNT) on depression (DEP) through fear of own negative evaluation (FONE) and fear of parental negative evaluation (FPNE) for South Asian participants. **(B)** Mediation of social norm transgression (SNT) on depression (DEP) through fear of own negative evaluation (FONE) and fear of parental negative evaluation (FPNE) for White participants. ^*^p < 0.05, ^**^p < 0.01, and ^***^p < 0.001.

For South Asian participants, the estimate of the indirect effect through fear of parental negative evaluation also fell within a positive confidence interval [*b* = 1.55, *SE* = 0.52, 95% CI (0.60, 2.67)]. In contrast, the estimate of the indirect effect through fear of own negative evaluation fell within a confidence interval that was neither positive or negative, crossing over zero [*b* = 0.54, *SE* = 0.34, 95% CI (−0.14, 1.21)]. As can be seen in Figure 1A, these results suggest that for South Asians, transgressing a social norm may be associated with depression in part through an increase in fear of parental negative evaluation but not through fear of own negative evaluation.

For the White participants, the estimate of the indirect effect through fear of parental negative evaluation fell within a confidence interval that was neither positive or negative, crossing over zero [*b* = 1.06, *SE* = 0.64, 95% CI (−0.19, 2.32)]. In contrast, the estimate of the indirect effect through fear of own negative evaluation fell within a positive confidence interval [*b* = 0.73, *SE* = 0.27, 95% CI (0.23, 1.29)]. As can be seen in Figure 1B, these results suggest that for White participants, transgressing a social norm may be associated with depression in part through an increase in fear of own negative evaluation but not through fear of parental negative evaluation.

Thus, for the South Asian participants fear of parental negative evaluation partially mediated the association between transgressing a social norm and depression whereas for the White participants fear of own negative evaluation partially mediated the association, providing support for Hypothesis 2. Additionally, the total effects of the mediation models increased average depression scores from the mild to moderate clinical ranges for South Asians participants (11.29 to 17.96) and for White participants (10.14 to 14.69).

#### Anxiety

The estimate reflecting the direct effect of a social norm transgression on anxiety for the South Asian participants fell within a positive confidence interval [*b* = 5.27, *SE* = 0.87, *p* < 0.001, 95% CI (3.55, 6.99)] as did that estimate for the White participants [*b* = 3.58, *SE* = 0.86, *p* < 0.001, 95% CI (1.89, 5.27)]. These results suggest that as perceptions of transgressing a social norm increase so may anxiety, providing support for Hypothesis 1.

**Figure 2:**
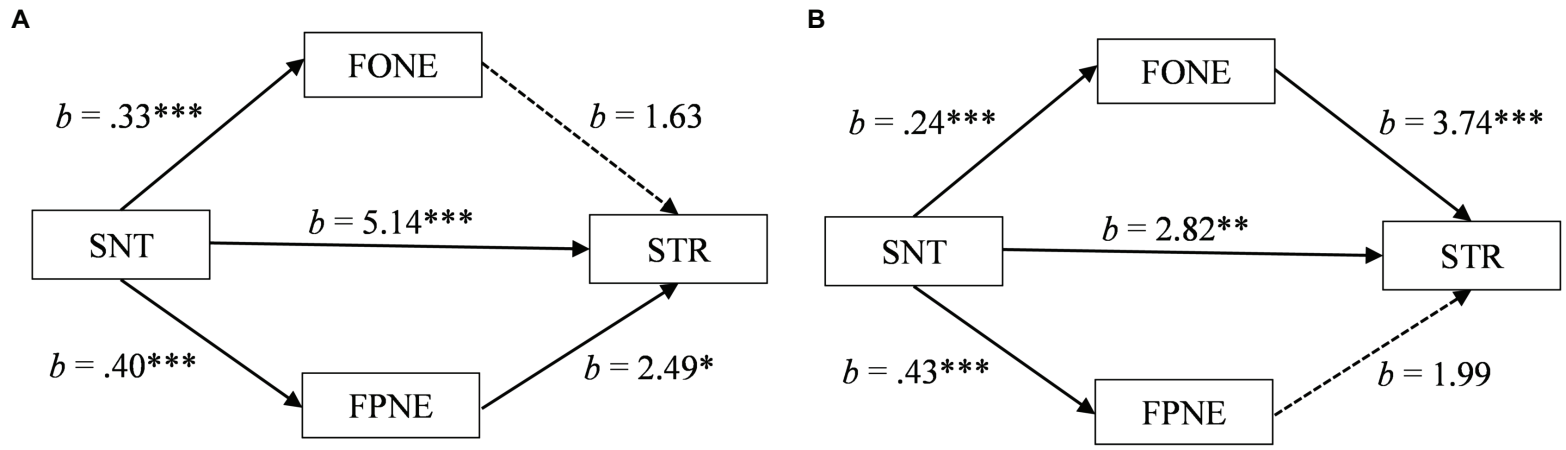
**(A)** Mediation of social norm transgression (SNT) on anxiety (ANX) through fear of own negative evaluation (FONE) and fear of parental negative evaluation (FPNE) for South Asian participants. **(B)** Mediation of social norm transgression (SNT) on anxiety (ANX) through fear of own negative evaluation (FONE) and fear of parental negative evaluation (FPNE) for White participants. ^*^p < 0.05, ^**^p < 0.01, and ^***^p < 0.001.

For the South Asian participants, the estimate of the indirect effect through fear of parental negative evaluation also fell within a positive confidence interval [*b* = 1.19, *SE* = 0.47, 95% CI (0.32, 2.17)]. In contrast, the estimate of the indirect effect through fear of own negative evaluation fell within a confidence interval that was neither positive or negative, crossing over zero [*b* = 0.21, *SE* = 0.29, 95% CI (−0.39, 0.78)]. As can be seen in Figure 2A, these results suggest that for South Asians, transgressing a social norm may be associated with anxiety in part through an increase in fear of parental negative evaluation but not through fear of own negative evaluation.

For the White participants, the estimate of the indirect effect through fear of parental negative evaluation also fell within a positive confidence interval [*b* = 1.29, *SE* = 0.54, 95% CI (0.25, 2.35)] and the estimate of the indirect effect through fear of own negative evaluation fell within a confidence interval that was neither positive or negative, crossing over zero [*b* = 0.36, *SE* = 0.21, 95% CI (−0.02, 0.80)]. As can be seen in Figure 2B, these results suggest that for White participants as well, transgressing a social norm may be associated with anxiety in part through an increase in fear of parental negative evaluation but not through fear of own negative evaluation.

Thus, for both the South Asian participants and White participants fear of parental negative evaluation partially mediated the association between transgressing a social norm and anxiety. This mediation provides partial support for Hypothesis 2, that for those from more collectivistic cultures, fear of negative evaluation of close others would mediate the association between social norm transgressions and psychological distress. However, the mediation does not provide support for the second part of Hypothesis 2, that for those from less collectivistic cultures, fear of negative evaluation of the self would mediate the association between social norm transgressions and psychological distress. Additionally, the total effects of the mediation models increased average anxiety scores from the moderate to severe clinical range for South Asian participants (10.67 to 17.34) and from the mild-to-moderate clinical range for White participants (8.90 to 14.13).

#### Stress

The estimate reflecting the direct effect of a social norm transgression on stress for the South Asian participants fell within a positive confidence interval [*b* = 5.14, *SE* = 0.85, *p* < 0.001, 95% CI (3.47, 6.81)] as did that estimate for the White participants [*b* = 2.82, *SE* = 0.90, *p* < 0.01, 95% CI (1.04, 4.59)]. These results suggest that as perceptions of transgressing a social norm increase so may stress, providing support for Hypothesis 1.

**Figure 3:**
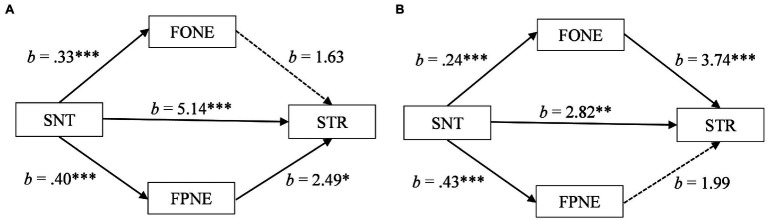
**(A)** Mediation of social norm transgression (SNT) on stress (STR) through fear of own negative evaluation (FONE) and fear of parental negative evaluation (FPNE) for South Asian participants. **(B)** Mediation of social norm transgression (SNT) on stress (STR) through fear of own negative evaluation (FONE) and fear of parental negative evaluation (FPNE) for White participants. ^*^p < 0.05, ^**^p < 0.01, and ^***^p < 0.001.

For South Asian participants, the estimate of the indirect effect through fear of parental negative evaluation also fell within a positive confidence interval [*b* = 0.99, *SE* = 0.44, 95% CI (0.21, 1.93)]. In contrast, the estimate of the indirect effect through fear of own negative evaluation fell within a confidence interval that was neither positive or negative, crossing over zero [*b* = 0.54, *SE* = 0.29, 95% CI (−0.02, 1.11)]. As can be seen in Figure 3A, these results suggest that for South Asians, transgressing a social norm may be associated with stress in part through an increase in fear of parental negative evaluation but not through fear of own negative evaluation.

For the White participants, the estimate of the indirect effect through fear of parental negative evaluation fell within a confidence interval that was neither positive or negative, crossing over zero [*b* = 0.86, *SE* = 0.54, 95% CI (−0.24, 1.93)]. In contrast, the estimate of the indirect effect through fear of own negative evaluation fell within a positive confidence interval [*b* = 0.89, *SE* = 0.26, 95% CI (0.40, 1.42)]. As can be seen in Figure 3B, these results suggest that for White participants, transgressing a social norm may be associated with stress in part through an increase in fear of own negative evaluation but not through fear of parental negative evaluation.

Therefore, for the South Asian participants fear of parental negative evaluation partially mediated the association between transgressing a social norm and stress whereas for the White participants fear of own negative evaluation partially mediated the association, providing support for Hypothesis 2. Additionally, the total effects of the mediation models increased average stress scores from the normal to mild clinical ranges for South Asians participants (11.31 to 17.98) and for White participants (11.09 to 15.66).

### Discussion

The aim of Study 2 was to examine the association between social norm transgressions and psychological distress, and cultural differences in the type of fear of negative evaluation that may mediate that association. Results provided support for the association between social norm transgressions and psychological distress. Results also suggested that for those from more collectivistic cultures increased fear of negative evaluation of close others partially mediates that association whereas for those from less collectivistic cultures increased fear of negative evaluation of the self partially mediates that association. The one exception was anxiety, for which cultural differences were not observed. For people from both more and less collectivistic cultures, only fear of negative evaluation of close others partially mediated the association between social norm transgressions and anxiety.

## General Discussion

### Mental Health

The results of the present research suggest an association between social norm transgressions and poor mental health. Specifically, the results suggest that social norm transgressions are positively associated with depression, anxiety, and stress. The results of the present research also suggest that fear of negative evaluation may play a role in the association between social norm transgressions and poor mental health. Specifically, it was observed that social norm transgressions were associated with increased fear of negative evaluation which, in turn, was associated with psychological distress. Furthermore, we observed that average scores on all three measures of psychological distress increased to a more severe clinical range (Lovibond and Lovibond, 1995) as a result of social norm transgressions and increased fear of negative evaluations. Depression scores increased from the mild to moderate clinical ranges, and stress scores increased from the normal to mild clinical ranges for all participants. Anxiety scores increased from the moderate to severe clinical range for the South Asian participants and from the mild to moderate clinical range for the White participants. Therefore, the results suggest that transgressing social norms and a resulting fear of being negatively evaluated can have a significant negative impact on one’s mental health. These observed results align with existing theorizing about the association between social norm transgressions and poor intrapersonal outcomes (e.g., McGraw, 1987; Giguère et al., 2014; Vaswani et al., 2021). However, the results of the present research provide novel empirical evidence for an association between social norm transgressions and poor mental health.

The notion that transgressing prevalent social norms is associated with poor mental health is theoretically assumed. However, empirical evidence for that assumption has previously not been established. The results of the present research provide a novel empirical basis for that assumption and build upon the literature aiming to understand and explain the consequences of social norm transgressions for mental health. The observed results support the assumption that transgressing prevalent social norms can have negative consequences for one’s mental health, particularly in terms of depression, anxiety, and stress. Alternatively, adhering to prevalent social norms can be beneficial to one’s mental health.

### Social Norms

Adherence to prevalent social norms helps to maintain agreed upon standards over how to think, feel, and behave, whereas social norm transgressions threaten such standards. Social responses such as cues of acceptance for social norm adherence and cues of rejection for social norm transgressions are common outcomes that motivate social norm adherence and deter transgressions (Festinger, 1950; Turner, 1991; Cialdini and Trost, 1998). Thus, social acceptance and social rejection can help maintain desirable social standards. Fear of negative evaluation is closely related to social rejection operating as a mechanism to deter social norm transgressions because norm transgressors may be concerned about others’ perceptions of them and resulting negative social consequences. We examined fear of negative evaluation as an outcome of social norm transgressions and, in turn, a predictor of poor mental health. The mediating role of fear of negative evaluation observed in the present research can be understood as a cognitive process that partially explains the association between social norm transgressions and poor mental health. Specifically, after social norm transgressions, concerns over negative evaluations were associated with poor mental health. Those concerns can act as a self-regulatory mechanism encouraging social norm adherence and discouraging social norm transgressions, thereby protecting mental health, and helping to maintain desirable social standards.

Concerns over negative responses from others can at times be harmful. For instance, concerns over being negatively evaluated can result in inappropriate behavior aimed at evaluation avoidance (e.g., self-sabotage, attempting to hide social norm transgressions, social withdrawal; see Giguère et al., 2016). However, fear of negative evaluation can also serve a functional purpose in regulating inappropriate behavior. For instance, the results of the present research suggest that making salient to potential norm transgressors that people will think poorly of them or their close others if they transgress social norms may motivate them to refrain from engaging in norm transgressing behavior. Aligning with the process of social learning based upon contingent outcomes of one’s actions (Bandura, 1977), negative evaluations of one’s norm transgressing behavior can act as positive punishment and result in subsequent decreases in such behavior. As a result, potential norm transgressors could avoid experiencing negative social responses from others (e.g., negative evaluations, dislike, disapproval, social rejection) and negative intrapersonal responses (e.g., judging oneself or one’s behavior as being immoral, feeling guilt and/or shame, experiencing depression, anxiety, and/or stress), and constructive social norms could be maintained.

### Fear of Negative Evaluation of Oneself Versus Close Others as a Function of Culture

The results of the present research also suggest a cultural difference in how fear of negative evaluation mediates the association between social norm transgressions and mental health. We observed that for people from more collectivistic cultures, the association between social norm transgressions and psychological distress occurred, in part, through an increase in fear of negative evaluation of close others. In contrast, for people from less collectivistic cultures, the association between social norm transgressions and psychological distress occurred, in part, through an increase in fear of negative evaluations of oneself. The one exception to this general pattern was for psychological distress experienced as anxiety. This exception may be because anxiety includes a concern over future events (e.g., exposure to certain objects, situations and places, people, and others’ evaluations). It may be that after having transgressed social norms, people are aware of how they have or will respond to negative evaluations of them. However, they may be in the dark as to how those close to them may be impacted by and respond to negative evaluations from others. That uncertainty may be driving fear of evaluation of close others rather than fear of evaluation of the self to mediate the association between social norm transgressions and anxiety for people from both more and less collectivistic cultural groups.

The observed overall cultural difference in the mediating role of fear of negative evaluation exemplifies that people with different knowledge systems, which are learned through their environments and experiences, and over generations, result in different ways of thinking (Hong, 2009). This cross-cultural perspective is in contrast to assuming that human thought, emotion, and behavior are universal phenomena. In the present research, people from more collectivistic cultures who tend to be closely connected to others and group dependent experienced psychological distress, in part, due to a concern over how their inappropriate behavior would impact close others. In contrast, people from less collectivistic cultures who tend to be less closely connected to others and less group dependent experienced psychological distress, in part, due to a concern over how their inappropriate behavior would impact themselves. These results add novel empirical evidence to existing literature on cultural differences in concerns over social image (e.g., Rodriguez Mosquera et al., 2002, 2008, 2014; Uskul et al., 2012) and psychological phenomena more generally (e.g., decision-making, conformity, attribution, parenting; see Heine, 2012).

### Limitations and Future Research Directions

One limitation of the present research is that the sample used in Study 1 to determine what events are commonly reported as social norm transgressions was a young adult student sample (on average 19 years old), and the sample used in Study 2 to test the hypotheses in that emerging domain was a community sample with an older average age (on average 29 years old). Social norm transgressions in the domain of partying may be experienced differently by individuals at these two different ages. Thus, future research should examine the associations between social norm transgressions, resulting fear of negative evaluation of oneself and close others, and psychological distress in the domain of partying using a student sample. Additionally, what constitutes common social norm transgressions among slightly older community samples should also be examined, and the associations of interest in the present research should be replicated in that emerging domain. A second limitation is that attempting to determine what events are commonly reported as social norm transgressions in Study 1 involved asking participants to recall events they would not want their parents to know about rather than norm transgressions more broadly. As such, the results of the present research should not be generalized to social norm transgressions too broadly, and future research should examine the associations of interest in the present research using a broader range of norm transgressions.

Another limitation of the present research is that the results are drawn from cross-sectional data limiting causal conclusions to be drawn. Furthermore, all measures used in the present research were self-report measures which can result in social desirability bias such that participants may be responding in a manner to present themselves more favorably. Additionally, measures used in the present research required participants to recall past events and their feelings immediately after those events which can result in recall bias wherein participants’ recollection may not be entirely accurate. Thus, future studies examining the association between social norm transgressions, psychological distress, and the mediating role of different types of fear of negative evaluation cross-culturally should be conducted using experimental or longitudinal study designs and include observable outcomes to allow for more definitive causal conclusions to be drawn. Additionally, the observed results should not be generalized to cultural differences between cultural groups other than those used in the present research. Rather, future research should determine whether the observed results generalize to other groups that differ in levels of collectivism.

## Conclusion

The present research aimed to examine the association between social norm transgressions and psychological distress, and cultural differences in the type of fear of negative evaluation that may mediate that association (i.e., fear of negative evaluation of close others for those from more collectivistic cultures and fear of negative evaluation of the self for those from less collectivistic cultures). Overall, the results suggest that social norm transgressions are associated with greater psychological distress, in particular, depression, anxiety, and stress. The results also suggest that this association appears to be driven by fears of being negatively evaluated by others for people from less collectivistic cultures, while it appears to be driven by fears that close others may be negatively evaluated for people from more collectivistic cultures.

## Data Availability Statement

The raw data supporting the conclusions of this article will be made available by the authors, without undue reservation.

## Ethics Statement

The studies involving human participants were reviewed and approved by University of Guelph Research Ethics Board. The patients/participants provided their written informed consent to participate in this study.

## Author Contributions

MV and BG contributed to conception and design of the study. MV performed data collection, the statistical analysis, and wrote the first draft of the manuscript. All authors contributed to the manuscript revision, read, and approved the submitted version.

## Funding

This research was supported by a Social Sciences and Humanities Research Council of Canada (SSHRC) doctoral scholarship to MV (#767-2017-2148), and a research grant to BG and IN-C (#430254).

## Conflict of Interest

The authors declare that the research was conducted in the absence of any commercial or financial relationships that could be construed as a potential conflict of interest.

## Publisher’s Note

All claims expressed in this article are solely those of the authors and do not necessarily represent those of their affiliated organizations, or those of the publisher, the editors and the reviewers. Any product that may be evaluated in this article, or claim that may be made by its manufacturer, is not guaranteed or endorsed by the publisher.

## Footnotes

^1^The measure of collectivism used in the present study can be divided into two subscales of horizontal and vertical collectivism. We conducted exploratory analyses comparing the two groups on each subscale. Focusing on vertical collectivism (Cronbach’s *α* South Asian = 0.78, White = 0.63), these exploratory analyses suggest that the South Asian group on average scored higher (*M* = 3.92, *SD* = 0.68) than the White group (*M* = 3.75, *SD* = 0.58), *t*(408) = −2.61, *SE* = 0.06, *p* < 0.01, 95% CI for mean difference [−0.28, −0.04]. Average scores for the South Asian group (*M* = 3.87, *SD* = 0.76) and the White group (*M* = 3.79, *SD* = 0.67) did not statistically significantly differ on the horizontal collectivism subscale (Cronbach’s *α* South Asian = 0.79, White = 0.73), *t*(408) = −1.18, *SE* = 0.07, *p* = 0.240, 95% CI for mean difference [−0.22, 0.06].

^2^We also assessed individualism as part of a broader study that the present research was situated within. It should be noted that Cronbach’s alphas were lower for the measure of individualism than collectivism, and did not reach the commonly agreed standard for acceptability for the White sample (Cronbach’s *α* South Asian = 0.75, Cronbach’s *α* White = 0.63). As with collectivism, on average the South Asian group (*M* = 3.70, *SD* = 0.62) scored higher on the measure of individualism than the White group (*M* = 3.52, *SD* = 0.50), *t*(408) = −3.28, *SE* = 0.06, *p* < 0.01, 95% CI for mean difference [−0.29, −0.07]. We did not examine moderation by individualism of the mediations because we had not planned to examine it and we did not have sufficient power to examine it.

^3^Moderated mediations for each outcome variable pooling the two groups and using group as the moderator are not being reported because of insufficient statistical power. However, linear models exploring this option revealed the same pattern of results as the mediation models for the two groups separately being reported, albeit with the upper level interaction terms not reaching commonly agreed criteria for statistical significance.

^4^Moderated mediations for each outcome variable pooling the two groups and using collectivism as the moderator was not conducted. Those analyses were not originally planned, and the sample size did not provide sufficient power to examine them. At the request of reviewers, we conducted exploratory analyses using collectivism (COL) as a moderator of the mediations. The models revealed that the interaction terms did not reach commonly agreed criteria for statistical significance (Depression: COL*FONE *p* = 0.897, COL*FPNE *p* = 0.209; Anxiety: COL*FONE *p* = 0.215, COL*FPNE *p* = 0.846; Stress: COL*FONE *p* = 0.972, COL*FPNE *p* = 0.340).
